# The Treatment of Eclampsia

**Published:** 1907-04

**Authors:** 


### THE TREATMENT OF ECLAMPSIA.

Evans writes an important paper on this subject in the British Medical Journal of November 3, 1906. He favors the more conservative method of dealing with eclampsia, considering that mutilating operations for effecting delivery should be reserved for the more serious cases in which in spite of treatment the danger to mother or child is very great.

In the severe hepatic form, with profound coma, small, high tension pulse, and scanty, blood-stained urine, delivery should be accomplished as soon as possible, if necessary by surgical means, under deep anesthesia.

In the nephritic form general treatment may be relied upon in the interests of the child up to the thirty-sixth week, the mortality of eclampic children being very high. In cases occurring after the thirty-sixth week the condition of the child should indicate the method of delivery to be adopted, except in those cases in which the mother's condition rapidly grows worse, as indicated by coma and the condition of the pulse tension.

Immediate operative treatment is indicated in the fulminant cases after the thirty-sixth week in the interests of the child, and in such cases occurring at an earlier period of pregnancy, in the interests of the mother.

One important joint in the early treatment of the eclampic condition is that absolute quiet of the patient must be secured. All manipulation of the patient for any purpose by the physician or nurse should be reduced to the minimum. Deep anesthesia should be secured before any obstetric examination, or to permit the administration of enemas, the passage of the stomach pump, etc.

In the large proportion of cases delivery can be effected by manual dilatation and the early application of forceps, or by version. In cases in which the cervix is long and the internal os not taken up, incision of the cervix is the most satisfactory method of effecting an entrance into the uterus. This can be accomplished according to the method of Bumm, by stripping up the bladder and cutting the cervix in the median line, or by Duhrssen's method of cutting both the anterior and posterior lips of the cervix after pushing up the bladder and peritoneum. Statistics of both methods show but little higher mortality than the spontaneous delivery.

The use of the Bossi metallic dilator, though much has been claimed for it, is not to be recommended unless the cervix is at least fairly well taken up.

Hydrostatic dilators play no role in the treatment of eclampsia, in the author's opinion, as no artificial dilating force should be employed upon the os or cervix, except under the deepest anesthesia, which should not be prolonged.

Cæsarian section by the abdominal route is but rarely indicated in eclampsia, and then only to save the child when the mother's condition is hopeless; the mortality of the operation in eclampsia, according to the statistics of Pollock and Boldt, being well over 50 per cent.

In the general treatment of eclampsia there is not a drug used nor a procedure employed on theoretical grounds, or as a result of experience, that has not as many enemies as friends.

Chloral and chloroform seem to be sheet-anchors in the treatment of eclampsia. The former should be used early and in large doses at intervals of four hours. Chloroform should be employed dur ing all manipulations of the patient, but not during the convulsions, as it is then only productive of harm, oxygen inhalations being preferable.

Opium and morphine have been used considerably since revived by Veit; but while they act favorably upon the nervous system and reduce the arterial tension, they certainly tend to interfere with the process of elimination, prolong the coma, and act unfavorably upon the child when employed for any length of time.

Veratrum viride is employed as a specific by many, believing that it promotes elimination by reducing the arterial tension. It certainly reduces the tension, but at too great a cost, as it has been blamed for many cases of heart failure where it has been employed. It must be carefully used in conjunction with camphor or caffeine to get the best results.

Bleeding is advocated by many as relieving the heart and reducing the toxemia. Venesection is of value in plethoric cases, but should not be employed in asthenic cases.

Subcutaneous and intravenous salines are supposed to act favorably, by diluting the toxins and favoring elimination.

Mace and Pierra have demonstrated that injections of normal saline solution result in the volume of urine passed being diminished and becoming more concentrated, resulting in a lower freezing point from the greater molecular concentration. They demonstrate that in eclampsia there is a notable pathological increase in the quantity of sodium chloride in the blood. For these reasons they conclude that salines should not be employed, and have recommended pure water injections and a water diet during the first twenty-four hours.

Subcutaneous and intravenous salines are, according to many authors, contraindicated when the arterial tension is high, the edema marked, and the kidney function much impaired.

Liepmann claims to have demonstrated that the toxin of eclampsia is of an albuminous nature, and that it arises in the placenta, passes over into the maternal organism, and is there fixed, chiefly by the cells of the liver and of the brain. The organs principally affected by the toxin are the brain, liver, and kidneys.

On these grounds Liepmann makes a strong argument for immediate delivery after the first convulsion, preferring manual dilatation and the use of the forceps if possible, and the employment of the Bossi dilator or anterior hysterotomy if the cervix is not taken up. The subsidiary treatment, as he terms it, of eclampsia is directed according to which organ shows the greatest damage—the kidneys, the heart, or the brain.

When the urine is loaded with albumin, casts, and blood Liepmann employs saline transfusion, venesection, and administration of diuretin, digitalis, and camphor in the form of a powder three times daily. When the heart is weak and the pulse is small and irregular he employs caffeine and camphor subcutaneously in large doses. When the brain bears the brunt of the attack, as is manifested by coma, superficial breathing (due to paralysis of the respiratory centers), lung edema, and severe convulsions, he employs artificial respiration almost continuously with intervals of half an hour, in which he recommends stimulation by means of slapping the patient with towels dipped in cold water. The convulsions he treats with large doses of chloral given by the rectum.

Morphine he only uses in the later stages as the patient is recovering, to control the restlessness. He considers artificial respiration is important to overcome the paralysis of the respiratory centers, and that the cold-water applications, acting reflexly through the vasomotor system on the edema of the lungs, are extremely valuable. He condemns warm packs, as their action tends to concentrate the toxin. In this he is supported by Bumm and others. He expresses the hope that a specific in the form of a serum may yet be forthcoming.—Therapeutic Gazette, February, 1907.

